# Supplementary material for: Augmented manipulation ability in humans with six-fingered hands
Source: Nat Commun. 2019 Jun 3;10:2401. doi: 10.1038/s41467-019-10306-w (PMC6547737; doi:10.1038/s41467-019-10306-w)
Supplement: Supplementary file 8 — Supplementary Information [file 41467_2019_10306_MOESM8_ESM.pdf]

Supplementary Information for

“Augmented manipulation ability in humans with six fingered hands”

Mehring et al.

Supplementary note:

Exponential increase of possible movements with degrees-of-freedom (dof)

The range of possible movements increases exponentially with every dof. This can be illustrated by the following example: assume each finger can be in one of the three different movement states {rest, flexion, extension}. Then, if f fingers can be moved independently, there are 3^f different movement states. Hence, there is an exponential increase of possible movement states with the number of fingers. A similar argument could be applied to the number of joints or muscles. Likewise, in continuous space the volume of a mathematical space grows exponentially with every additional dimension.

Supplementary figures:

Subject P1

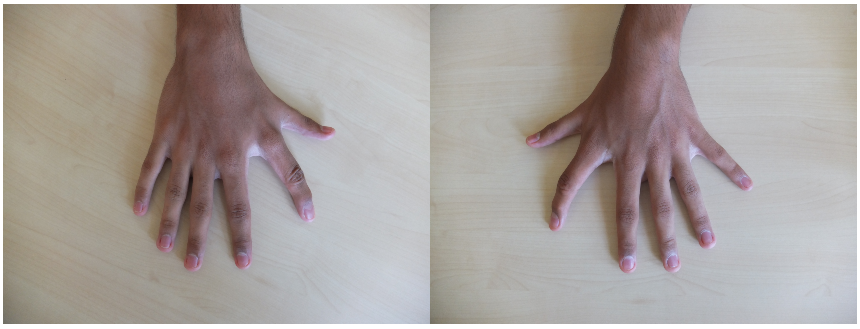

Subject P2

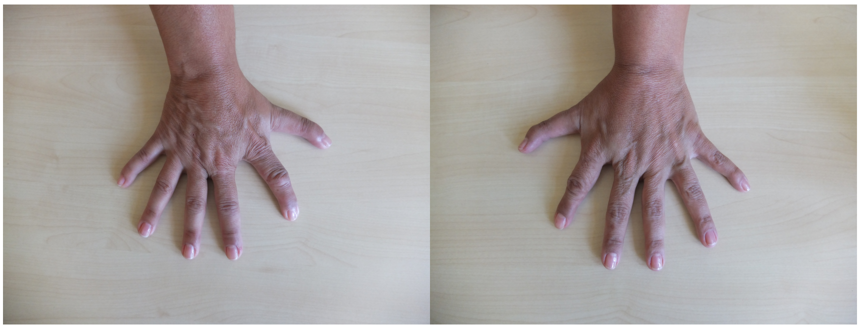

Supplementary Figure 1: Hands of subjects P1 and P2.

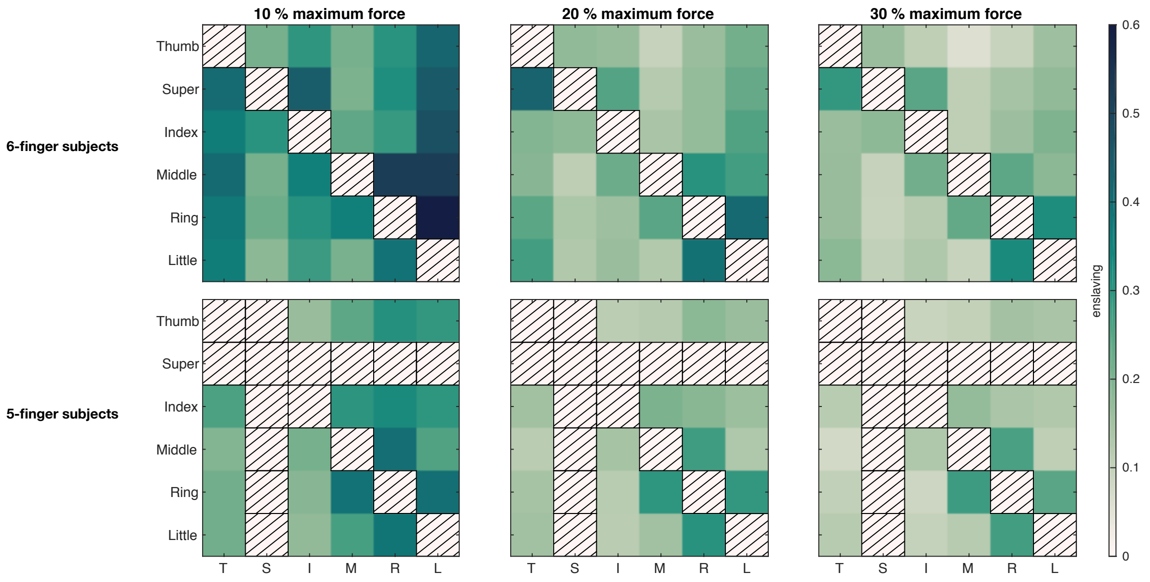

Supplementary Figure 2: Enslaving for 10%, 20% and 30% of maximal force for 5- and 6-fingered subjects. The instructed finger is shown on the y-axis, hence, each row shows the induced force relative to x% maximal force of the corresponding finger. Enslaving was similar to the enslaving observed for maximum force (Fig. 2D), in particular for 20% and 30% of maximum force (Pearson correlation coefficient between enslaving at x% and enslaving at maximum force; r=0,74, 0.90, 0.92 for 10%, 20% and 30% maximum force of 5-fingered subjects, N=20; r=0.30,0.61,0.72 for 6-fingered subjects, N=30). The enslaving magnitude was correlated between 5- and 6-fingered subjects across finger pairs available in both hands (Pearson correlation coefficient, r=0.44,0.69,0.74 for 10%,20%,30%, N=20).

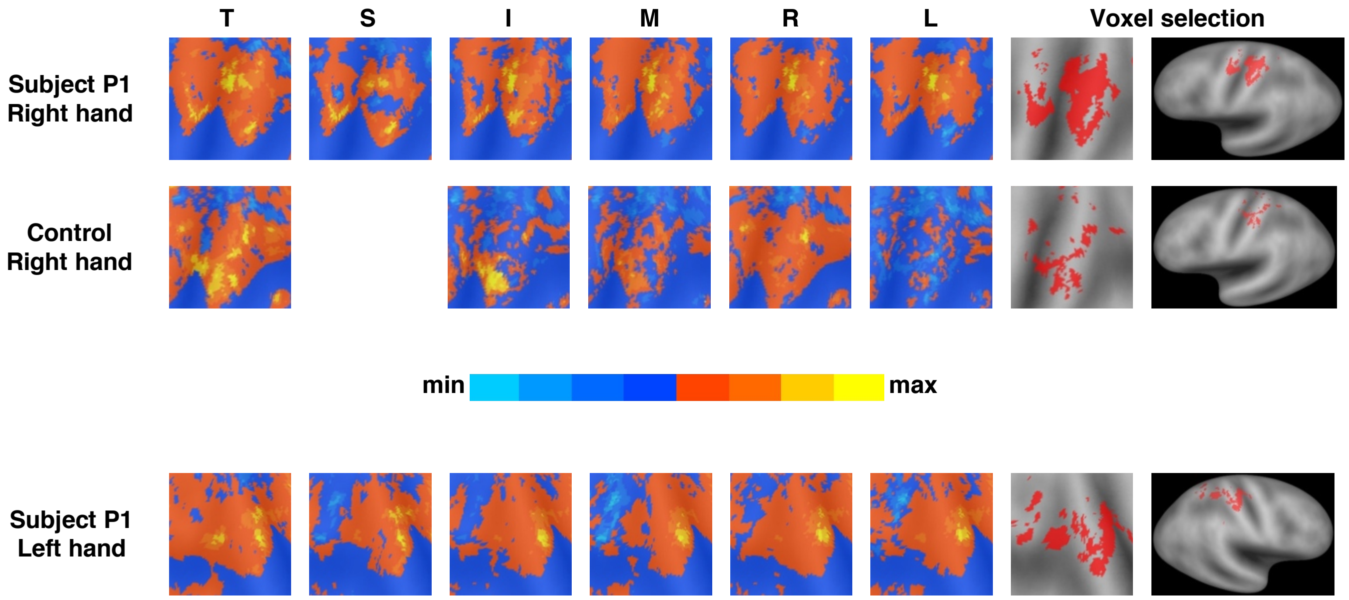

Supplementary Figure 3: fMRI activation patterns in right and left sensorimotor cortex of subject P1 during individual finger movements. The 2nd row shows the activation in left sensorimotor cortex of a 5-fingered control subject.

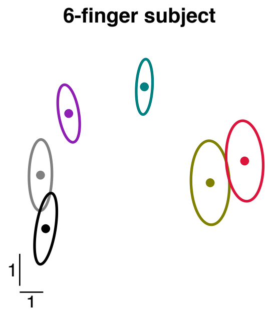

Supplementary Figure 4: Neural representation of fingers in right sensorimotor cortex (same as in Fig 2E for the left sensorimotor cortex).

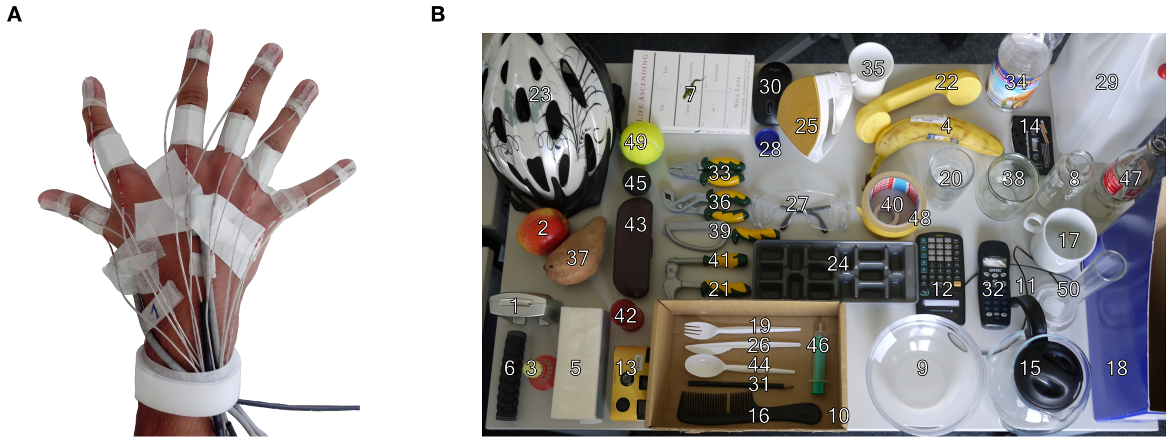

Supplementary Figure 5: (A) Recording of finger movements with accurate electromagnetic motion capture system (Polhemus Liberty 240/16-16) during the object manipulation and the common movement tasks. (B) Objects used in the object manipulation task (in the order as they were given to the subjects in the experiment): 1 alarm clock, 2 apple, 3 badminton shuttle, 4 banana, 5 blackboard eraser, 6 bicycle handle, 7 book, 8 glass bottle 0.25l, 9 bowl, 10 cardboard box, 11 cable, 12 calculator, 13 disposable camera, 14 audio cassette, 15 coffee jar, 16 comb, 17 cup, 18 folder, 19 fork, 20 glass, 21 hammer, 22 handset, 23 helmet, 24 Ice cube mold, 25 iron, 26 knife, 27 safety glasses, 28 bottle cap, 29 milk bottle 5l, 30 mouse, 31 pencil, 32 phone, 33 pincers, 34 plastic bottle 1l, 35 plastic cup, 36 wrench, 37 sweet potato, 38 jam jar, 39 saw, 40 medical tape, 41 screw driver, 42 pencil sharpener, 43 spectacle case, 44 spoon, 45 squash ball, 46 syringe, 47 coca cola bottle, 48 double faced adhesive tape, 49 tennis ball, 50 vase.

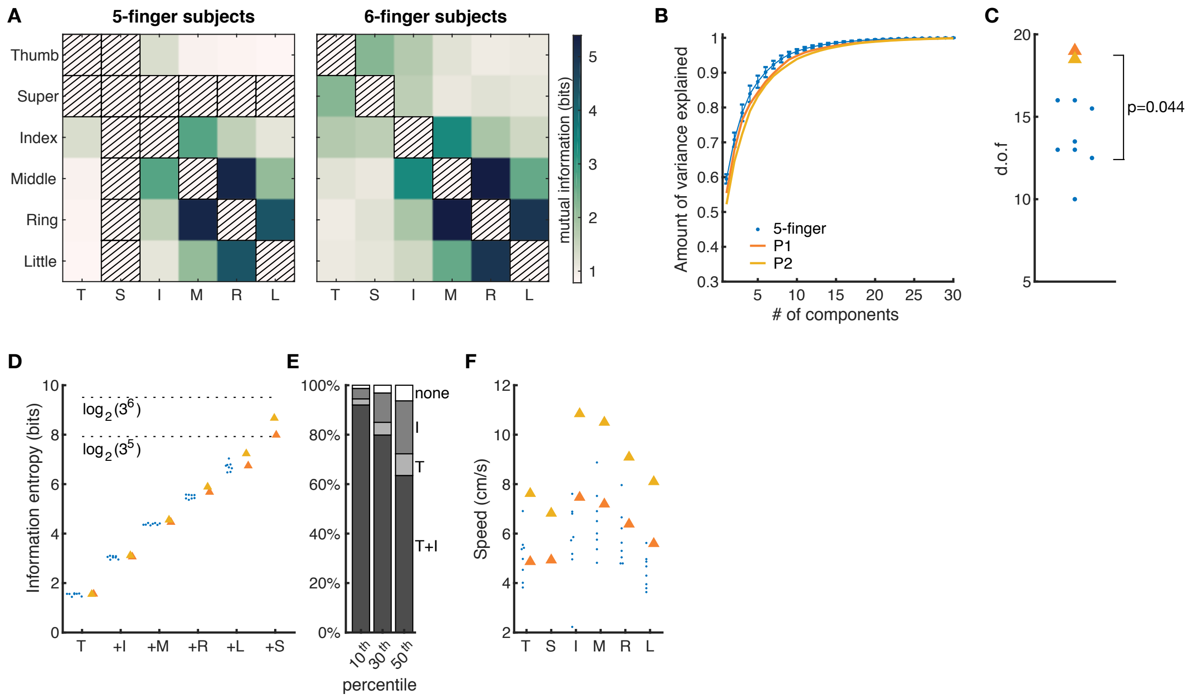

Supplementary Figure 6: This figure shows the same as figure 3A-F but for movements during common movement tasks. The movement tasks were carried out by two 6- and eight 5-fingered subjects. (A) Dependency between individual fingers quantified by the mutual information between the movements of pairs of fingers, with a value of 0 indicating complete independence between fingers and positive values an increasing dependency. Note that the mutual information is symmetric, i.e. $I(X,Y) = I(Y,X)$. (B) The cumulative amount of explained variance of hand movements as a function of an increasing number of principal components. Error bars depict SDs across subjects. (C) The number of effective dof (computed using the principle components, see Methods) was higher in 6-fingered than in 5-fingered subjects. (D) Information entropy of the discretized movements where each finger is either resting, flexing or extending. Entropy is shown for an increasing number of fingers, starting with thumb only ('T') and successively adding one finger (index 'I', middle 'M', ring 'R', little 'L' and supernumerary 'S'). Dotted lines indicate the theoretically maximum possible entropy for 5- and 6-fingered hands. (E) Percentage of times thumb and index finger ('T+I'), thumb only ('T'), index only ('I') were moving when the supernumerary finger moved. From left to right: different percentiles of the speed distribution were used as thresholds to separate rest from movement. (F) Median movement speed of individual fingers for 5- and 6-fingered subjects.

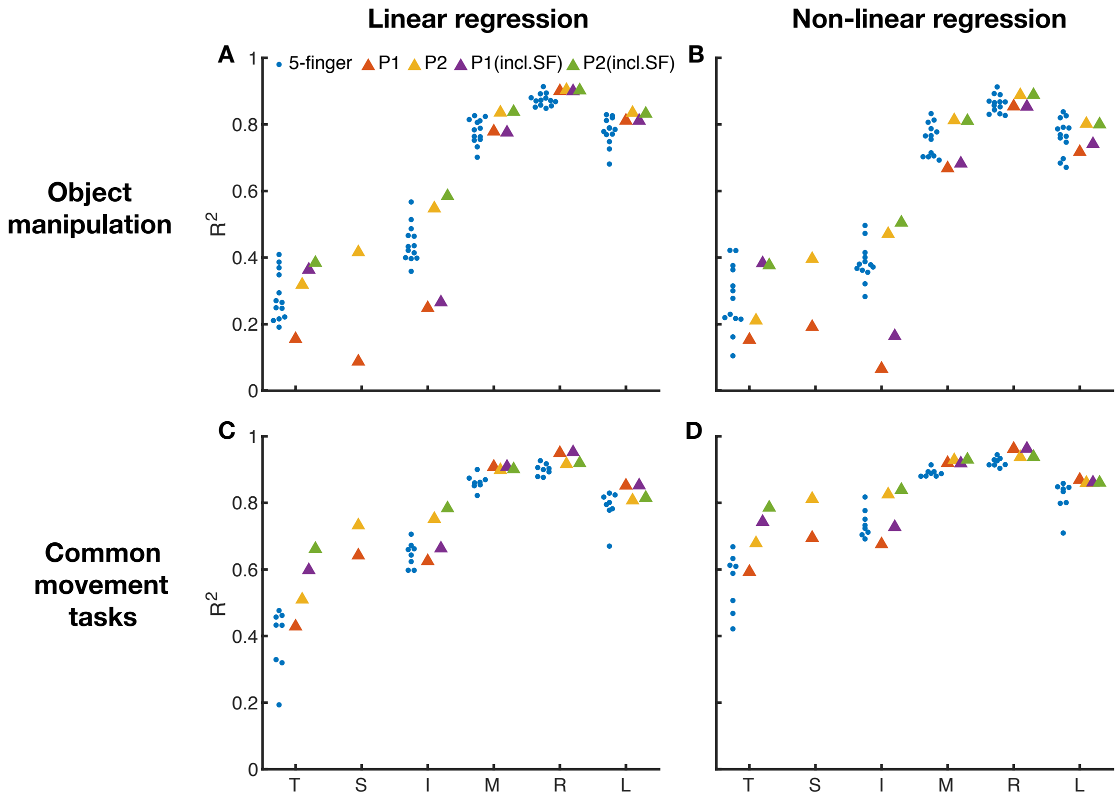

Supplementary Figure 7: Prediction of individual finger movement velocity from movement velocities of other fingers. Prediction was done using linear regression (A,C) and non-linear support vector (B,D) regression for the object manipulation (A,B) and the common movement tasks (C,D). The goodness of prediction was quantified by the coefficient of determination (R^2) between predicted and actual movement. Linear and non-linear models were fitted and evaluated on mutually exclusive data sets using cross-validation. See Methods for details. For 6-finger subjects the prediction was done including and excluding the supernumerary fingers. Without the supernumerary finger predictability for 5- and 6-finger subjects was similar. The predictability of the supernumerary finger is among the lowest across the fingers. It is similar to the thumb or index finger in object manipulation tasks while in common movement tasks it's slightly higher than the thumb and similar to the index finger.

Level	Performance Threshold Accuracy/Error (%)	Oscillation Frequency Range Hi/Low (Hz)	Speed Blocks
0	-/-	0.2/0.1	1
1	50/50	0.25/0.15	2
2	60/40	0.25/0.15	2
3	70/30	0.25/0.15	2
4	50/50	0.3/0.2	3
5	60/40	0.3/0.2	3
6	70/30	0.3/0.2	3
7	50/50	0.35/0.25	4
8	60/40	0.35/0.25	4
9	70/30	0.35/0.25	4
10	50/50	0.4/0.3	5
…	…	…	…
39	70/30	0.8/0.7	13

Supplementary Table 1: Parameters of different levels of the video game for 6-fingered subjects (see Fig. 3G,H).
