# Supplementary material for: Augmented manipulation ability in humans with six-fingered hands
Source: Nat Commun. 2019 Jun 3;10:2401. doi: 10.1038/s41467-019-10306-w (PMC6547737; doi:10.1038/s41467-019-10306-w)
Supplement: Supplementary file 9 — Description of Additional Supplementary Files [file 41467_2019_10306_MOESM9_ESM.docx]

Description of Additional Supplementary Files

**Supplementary Movie 1:** Handshape

**Supplementary Movie 2:** Force control task

**Supplementary Movie 3:** Pinch grip

**Supplementary Movie 4:** Object manipulation task

**Supplementary Movie 5:** Common movement tasks

**Supplementary Movie 6:** Video game for 6 fingers

**Supplementary Movie 7:** Various tasks with 6 fingers
