# Supplementary material for: Augmented manipulation ability in humans with six-fingered hands
Source: Nat Commun. 2019 Jun 3;10:2401. doi: 10.1038/s41467-019-10306-w (PMC6547737; doi:10.1038/s41467-019-10306-w)
Supplement: Supplementary file 11 — Reporting Summary [file 41467_2019_10306_MOESM11_ESM.pdf]

nature research

Corresponding author(s): Carsten Mehring and Etienne Burdet

Last updated by author(s): Carsten Mehring

Reporting Summary

Nature Research wishes to improve the reproducibility of the work that we publish. This form provides structure for consistency and transparency in reporting. For further information on Nature Research policies, see Authors & Referees and the Editorial Policy Checklist.

Statistics

For all statistical analyses, confirm that the following items are present in the figure legend, table legend, main text, or Methods section.

n/a	Confirmed	
☐	☒	The exact sample size (n) for each experimental group/condition, given as a discrete number and unit of measurement
☒	☐	A statement on whether measurements were taken from distinct samples or whether the same sample was measured repeatedly
☐	☒	The statistical test(s) used AND whether they are one- or two-sided<br>*Only common tests should be described solely by name; describe more complex techniques in the Methods section.*
☐	☒	A description of all covariates tested
☐	☒	A description of any assumptions or corrections, such as tests of normality and adjustment for multiple comparisons
☒	☐	A full description of the statistical parameters including central tendency (e.g. means) or other basic estimates (e.g. regression coefficient) AND variation (e.g. standard deviation) or associated estimates of uncertainty (e.g. confidence intervals)
☐	☒	For null hypothesis testing, the test statistic (e.g. F, t, r) with confidence intervals, effect sizes, degrees of freedom and P value noted<br>*Give P values as exact values whenever suitable.*
☒	☐	For Bayesian analysis, information on the choice of priors and Markov chain Monte Carlo settings
☒	☐	For hierarchical and complex designs, identification of the appropriate level for tests and full reporting of outcomes
☒	☐	Estimates of effect sizes (e.g. Cohen's d, Pearson's r), indicating how they were calculated

Our web collection on statistics for biologists contains articles on many of the points above.

Software and code

Policy information about availability of computer code

Data collection	Custom-made MATLAB scripts communicating with the API from the Polhemus liberty system. Psychophysics Toolbox Version 3 with custom-made MATLAB scripts. Custom program written in labview and experiment tasks written in Unity with a UDP protocol passing the data from labview. Custom-made python-based software for stimuli presentation inside the fMRI scanner.
Data analysis	Custom-made MATLAB scripts. SPM8 for fMRI data. OsiriX Dicom Viewer for the analysis of the anatomical MRI.

For manuscripts utilizing custom algorithms or software that are central to the research but not yet described in published literature, software must be made available to editors/reviewers. We strongly encourage code deposition in a community repository (e.g. GitHub). See the Nature Research guidelines for submitting code & software for further information.

Data

Policy information about availability of data

All manuscripts must include a data availability statement. This statement should provide the following information, where applicable:

- Accession codes, unique identifiers, or web links for publicly available datasets
- A list of figures that have associated raw data
- A description of any restrictions on data availability

The data are not available due to them containing information which could compromise research participant privacy and consent.

Field-specific reporting

Please select the one below that is the best fit for your research. If you are not sure, read the appropriate sections before making your selection.

☒ Life sciences ☐ Behavioural & social sciences ☐ Ecological, evolutionary & environmental sciences

For a reference copy of the document with all sections, see nature.com/documents/nr-reporting-summary-flat.pdf

Life sciences study design

All studies must disclose on these points even when the disclosure is negative.

Sample size	see Methods section
Data exclusions	see Methods section
Replication	N/A
Randomization	N/A
Blinding	N/A

Reporting for specific materials, systems and methods

We require information from authors about some types of materials, experimental systems and methods used in many studies. Here, indicate whether each material, system or method listed is relevant to your study. If you are not sure if a list item applies to your research, read the appropriate section before selecting a response.

Materials & experimental systems

n/a	Involved in the study	
☒	☐	Antibodies
☒	☐	Eukaryotic cell lines
☒	☐	Palaeontology
☒	☐	Animals and other organisms
☐	☒	Human research participants
☒	☐	Clinical data

Methods

n/a	Involved in the study	
☒	☐	ChIP-seq
☒	☐	Flow cytometry
☐	☒	MRI-based neuroimaging

Human research participants

Policy information about studies involving human research participants

Population characteristics	see Methods section
Recruitment	Polydacyly subjects were identified on the internet and then recruited by contacting them directly.<br>Five fingered subjects were recruited using flyers and lab websites.
Ethics oversight	see Methods section

Note that full information on the approval of the study protocol must also be provided in the manuscript.

Magnetic resonance imaging

Experimental design

Design type	task fMRI with block design
Design specifications	24 blocks of 20 seconds of finger tapping and 10 seconds of rest (4 repetitions per finger)
Behavioral performance measures	participants were trained on the finger tapping movements before entering the fMRI scanner

Acquisition

Imaging type(s): functional

Field strength: 7T

Sequence & imaging parameters: gradient echo EPI, resolution 1.3x1.3x1.3 mm3, slice thickness 1.3mm, no gap, matrix size 160x160, TR= 2s, FOV=210mm, TE=27ms, flip angle=75°, GRAPPA=2

Area of acquisition: Slices were manually placed over the central sulcus to cover the primary motor cortex

Diffusion MRI ☐ Used ☒ Not used

Preprocessing

Preprocessing software: SPM8

Normalization: analysis in native space

Normalization template: N/A

Noise and artifact removal: slice timing correction, spatial realignment, smoothing (FWHM=2mm) and temporal filtering

Volume censoring: N/A

Statistical modeling & inference

Model type and settings: single subject RSA

Effect(s) tested: N/A

Specify type of analysis: ☐ Whole brain ☒ ROI-based ☐ Both

Anatomical location(s): primary motor cortex

Statistic type for inference (See Eklund et al. 2016): peak-level inference

Correction: FWE

Models & analysis

n/a	Involved in the study
☒	☐ Functional and/or effective connectivity
☒	☐ Graph analysis
☒	☐ Multivariate modeling or predictive analysis
